# The EGFR-ADAM17 Axis in Chronic Obstructive Pulmonary Disease and Cystic Fibrosis Lung Pathology

**DOI:** 10.1155/2018/1067134

**Published:** 2018-01-09

**Authors:** Marta Stolarczyk, Bob J. Scholte

**Affiliations:** ^1^Cell Biology, Erasmus MC, Rotterdam, Netherlands; ^2^Pediatric Pulmonology, Erasmus MC, Rotterdam, Netherlands

## Abstract

Chronic obstructive pulmonary disease (COPD) and cystic fibrosis (CF) share molecular mechanisms that cause the pathological symptoms they have in common. Here, we review evidence suggesting that hyperactivity of the EGFR/ADAM17 axis plays a role in the development of chronic lung disease in both CF and COPD. The ubiquitous transmembrane protease A disintegrin and metalloprotease 17 (ADAM17) forms a functional unit with the EGF receptor (EGFR), in a feedback loop interaction labeled the ADAM17/EGFR axis. In airway epithelial cells, ADAM17 sheds multiple soluble signaling proteins by proteolysis, including EGFR ligands such as amphiregulin (AREG), and proinflammatory mediators such as the interleukin 6 coreceptor (IL-6R). This activity can be enhanced by injury, toxins, and receptor-mediated external triggers. In addition to intracellular kinases, the extracellular glutathione-dependent redox potential controls ADAM17 shedding. Thus, the epithelial ADAM17/EGFR axis serves as a receptor of incoming luminal stress signals, relaying these to neighboring and underlying cells, which plays an important role in the resolution of lung injury and inflammation. We review evidence that congenital CFTR deficiency in CF and reduced CFTR activity in chronic COPD may cause enhanced ADAM17/EGFR signaling through a defect in glutathione secretion. In future studies, these complex interactions and the options for pharmaceutical interventions will be further investigated.

## 1. Introduction

Airway epithelium, apart from providing a structural barrier against microbes and inhaled particles, also plays an active role in the first line of inflammatory responses [1], and thus emerged as a therapeutic target in chronic lung disease [2]. Airway epithelial cells respond dynamically to bacterial and viral infections [3–5] or inhaled noxious particles by transducing inflammatory signals [6, 7]. They produce cytokines, growth factors, and other inflammatory mediators to orchestrate epithelial repair and adaptation and recruit a range of inflammatory cells including neutrophils and macrophages [1, 8, 9]. Importantly, airway epithelial cells also crosstalk with the underlying connective tissue [10, 11].

This process normally leads to full resolution of inflammation and tissue damage. However, in chronic lung disease, exaggerated trans-signaling can lead to permanent changes of tissue structure and loss of lung function, chronic inflammation, and mucous metaplasia.

**Figure 1:**
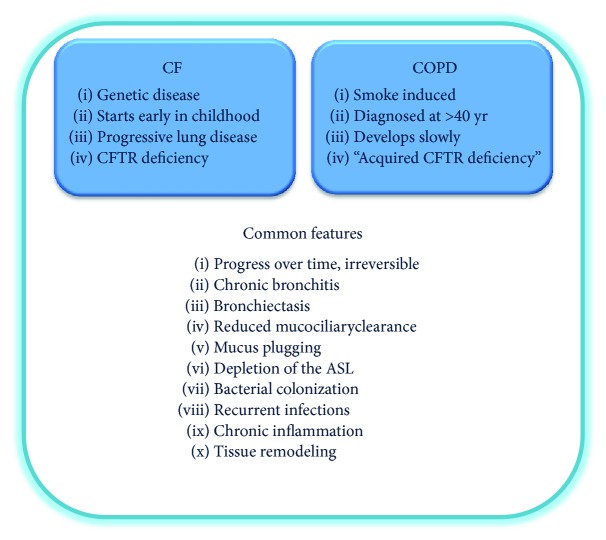
Common features of CF and COPD. CF and COPD lung disease share many common clinical manifestations despite the differences with respect to etiology. Mutations in the CFTR gene determine CF lung disease. Recently, CS has been shown to affect CFTR channel activity and this is recognized as a factor that may contribute to a CF-like phenotype in COPD. Thus, COPD has been recently described as “acquired CF.”

Despite their different etiology, cystic fibrosis (CF), a congenital abnormality caused by mutations in a chloride transporter, and chronic obstructive pulmonary disease (COPD), caused by chronic exposure to airborne irritants, share many common features in their pathology detailed in the paragraphs below (Figure 1) [12–14]. Both diseases are characterized by progressive and essentially irreversible lung damage, chronic bronchitis, bacterial colonization, reduced mucociliary clearance, and mucus plugging.

This leads to the question whether these diseases, while quite different in many aspects, share common molecular mechanisms and therapeutic targets (Figure 1) [15–17]. In this review, we focus on a signaling pathway called the EGFR/ADAM17 axis and its potential role in chronic lung disease, in particular CF and COPD. Similarly, but outside the scope of this review, other forms of chronic inflammatory lung disease, including asthma, can be added to this list.

**Figure 2:**
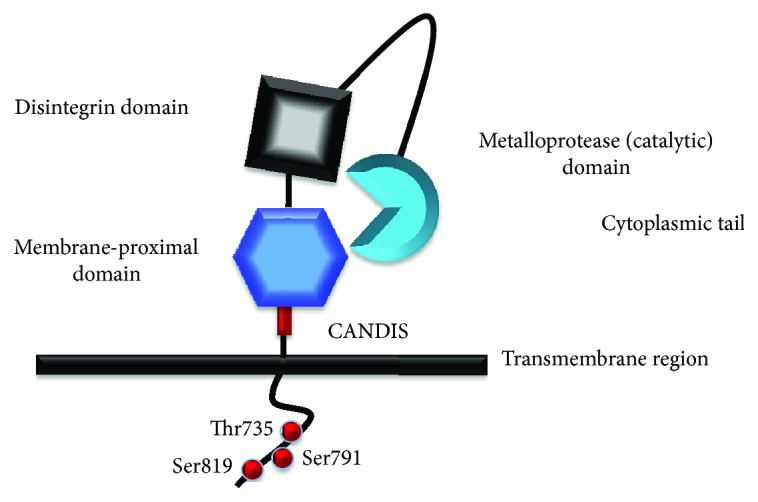
ADAM17 domain structure. ADAM17 is an atypical member of the ADAM family. It has additional disulfide bonds in the metalloprotease domain, and it lacks two calcium binding sites in the disintegrin domain. The membrane proximal domain (MPD), replacing the cysteine-rich and EGF-like domains, with a novel alpha/beta fold has a shorter cysteine-rich segment. The MPD has cysteine residues determining the ADAM17 conformation (open/closed) and ADAM17 protease activity (active/inactive switch). The MPD is in close proximity to the active site likely due to a C-shaped conformation of the extracellular part of mature ADAM17 [99]. ADAM17 lacks an EGF-like domain, so the MPD is followed by the juxtamembrane region “conserved ADAM17 dynamic interaction sequence” (CANDIS) involved in substrate recognition [162]. The trans-membrane region ends with a cytoplasmic tail with phosphorylation sites, which are likely important for ADAM17 activity and trafficking [97–101].

A disintegrin and metalloprotease 17 (ADAM17), a ubiquitous sheddase formerly known as TACE (Figure 2), that releases a broad spectrum of soluble biologically active ligands from airway epithelial and myeloid cells is recognized as an important transducer of airway epithelial cis- and trans-signaling [18]. As detailed below, ADAM17 interacts with the EGF receptor (EGFR), mainly through the shedding of EGFR ligands, and conversely, EGFR is required for ADAM17 activity, in a feedback signaling cascade labelled the EGFR/ADAM17 axis. This system is highly sensitive to various extracellular triggers, including cigarette smoke and bacterial toxins, resulting in the enhanced shedding of a large repertoire of growth factors, cytokines, and cytokine receptors that are substrates of ADAM17. This in turn leads to paracrine and autocrine receptor activation, which plays an important role in the resolution of airway inflammation and tissue damage.

**Figure 3:**
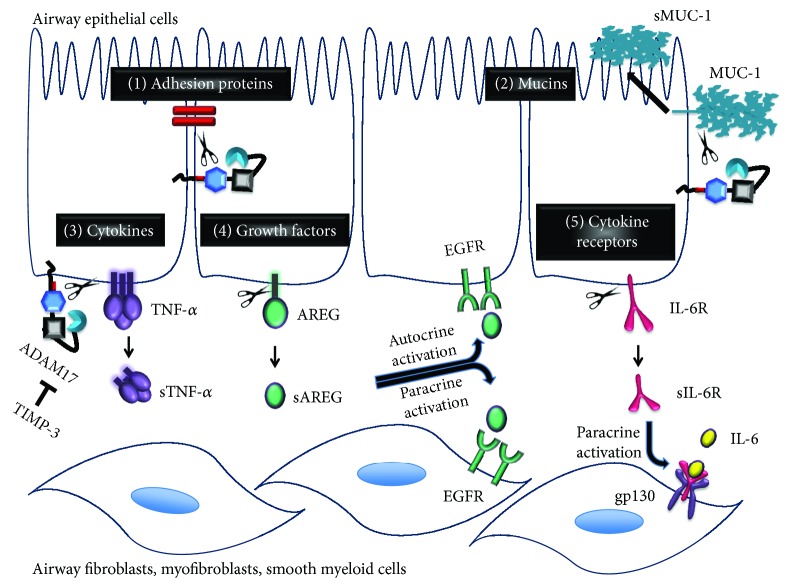
ADAM17 dependent paracrine and autocrine signaling. A disintegrin and metalloproteinase 17 (ADAM17), also known as a tumor necrosis factor-*α* converting enzyme (TACE), is involved in the immune defense mechanisms mediated by epithelial cells. ADAM17 releases extracellular domains of transmembrane proteins to produce soluble bioactive signaling proteins taking part in autocrine (activation of receptors within the same epithelial cell layer) and paracrine signaling (activation of cellular receptors on underlying neighboring cells, also termed transactivation). Among the ADAM17 substrates are (1) adhesion proteins (L-selectin, ICAM), (2) transmembrane mucins (MUC-1), (3) membrane-bound cytokines (TNF-*α*), (4) growth factors (AREG) and other ligands of EGFR (TGF-*α*, EREG, HB-EGF, and epigen), and (5) cytokine receptors (IL-6R, TNF-R). Ectodomain shedding provides the mechanism for autocrine and paracrine signaling. IL-6R shed from epithelial cells transactivates gp130 on the underlying myofibroblasts, whereas AREG shed from epithelial cells activates EGFR on epithelial cells or on the underlying fibroblasts. The ADAM17 inhibitor TIMP-3 inhibits the ADAM17 proteolytic activity.

In this review, we focus on the role of the epithelial EGFR/ADAM17 signaling pathway that transmits signals from luminal receptors towards underlying tissue (paracrine) and epithelial cells (autocrine) (Figure 3), affecting inflammation and remodeling. However, EGFR and ADAM17 are ubiquitously expressed, and also the expression of ADAM17 substrates is not limited to epithelial cells. Therefore, the complete signaling cascade also includes fibroblasts, smooth muscle cells, and myeloid cells.

The relative contribution of the different cell populations in the EGFR/ADAM17 signaling pathway in humans is still under investigation. Current knowledge is largely based on studies with conditional mutant murine models, in which ADAM17 can be selectively ablated in specific cell types. Knocking out ADAM17 in mesenchymal cells did not produce a detectable developmental phenotype, whereas epithelial ablation resulted in severe abnormalities [19]. Horiuchi et al. showed that the epithelial EGFR/ADAM17 axis plays a major role in tissue regeneration during colonic inflammation and that loss of myeloid expression does not have a major effect. However, myeloid ablation of ADAM17 does reduce the lethality of endotoxin shock induced by intraperitoneal injection [20]. While these studies give important insight in the basic architecture and activity of this system, due caution is required when applied to human tissues. In addition to obvious differences in architecture and function of human and mouse lungs, substrate specificity and expression patterns of members of the ADAM sheddase families and ligand interactions with receptors will differ.

We propose, based on evidence in the literature discussed below, that the molecular mechanism underlying the development of CF and COPD lung disease may involve abnormal activation of the epithelial ADAM17/EGFR axis. To put this in perspective, we first review CF and COPD pathology and the unmet need in clinical intervention. Next, we discuss the role of the EGFR/ADAM17 axis in lung pathology, the molecular mechanisms involved, and potential therapeutic strategies.

## 2. Cystic Fibrosis: A Congenital Lung Disease with an Early Onset

CF is an autosomal recessive lung disease caused by more than 2000 different mutations in the cystic fibrosis transmembrane conductance regulator (CFTR) gene [21] with approximately 80,000 patients worldwide. The most common CF mutation is the deletion of a phenylalanine at amino acid position 508 in the NBD1 domain of CFTR protein (F508del) [22]. The CFTR gene encodes a chloride channel mainly expressed in the apical membrane of secretory epithelial cells [23]. CFTR has a key role in maintaining ion and water homeostasis in secretory epithelia [23]. Mutations in CFTR cause a multiorgan CF disease affecting the lungs, intestine, pancreas, liver, sinuses, and reproductive organs [24]. However, under present treatment, the main morbidity and mortality among CF patients is due to lung malfunction; consequently, most available and experimental treatments aim to prevent the progression of CF lung disease [25]. CFTR is also involved in transport of other anions, for instance bicarbonate [26] and glutathione [27, 28]. Bicarbonate is crucial to normal expression of mucins that in CF remain aggregated and poorly solubilized [29]. Glutathione, as a natural antioxidant, reduces oxidative cellular stress [30].

### 2.1. CF Lung Disease: An Early Disorder of Distal Airways

Advances in imaging and monitoring of the respiratory system in infant patients have revealed that severe CF lung pathology starts early in childhood and progresses irreversibly over time [31–33]. This early onset of lung abnormalities includes bronchiectasis, diagnosed thickening and dilation of the bronchial walls, air trapping, and atelectasis (partial collapse of the lung) [34, 35]. These symptoms occur simultaneously with reduced mucociliary clearance and mucus plugging [36]. Quantitative and standardized tracking of early lung disease progression in infants with CT scans is pursued to advance the comparative analysis and provide the evaluation of the treatment [37, 38].

In a recent micro CT and histological study of end-stage CF lungs, the dilatation and obstruction of distal airways and a severe reduction in the number of functional terminal bronchioles were clearly documented for the first time. This confirms that obstruction and remodeling of peripheral airways is a prominent feature in CF lung disease and therefore are prime targets of experimental therapy [14]. CF mouse models [39] and large CF animal models, like pig [40] and ferret [41], are helpful in the investigation of the mechanism involved in this early onset of CF lung disease and intervention therapy, but all have considerable limitations due to species variation. In addition to state-of-the-art clinical and biomarker studies, there is an urgent need for the development of organotypic and personalized cell culture models in which the complex molecular and cellular interactions involved in CF lung pathology can be studied.

### 2.2. Mucociliary Transport

In healthy subjects, mucociliary clearance requires CFTR-dependent balanced fluid and proper mucus secretion from surface cells and subepithelial glands. However, high mucus viscosity in CF is caused by reduced CFTR-dependent bicarbonate secretion, required for proper expansion of secreted mucus molecules [42, 43]. Furthermore, CF patients have intrinsically impaired ciliary beat frequency (CBF), which is not only dependent on CFTR-mediated bicarbonate transport but also regulated by soluble adenyl cyclase (sAC) [44]. Together, this impairs effective clearance of bacteria and inhaled particles in CF lung [45, 46]. Importantly, reduced mucociliary clearance is also a feature of COPD, which may be in part related to reduced CFTR activity in smokers' lungs [47].

### 2.3. Bacterial Infection and Inflammation

Airway epithelial cell cultures from CF patients have reduced air-surface liquid (ASL) height, presumably due to defective CFTR-dependent fluid secretion [48]. Additionally, as a consequence of reduced bicarbonate secretion, CFTR deficiency abnormally acidifies ASL [49] which impairs bacterial killing [50, 51], inhibits the activity of ASL antimicrobials [52], and increases ASL viscosity of newborn CF piglets [45].

Impaired bacterial killing and mucociliary clearance in CF lungs together facilitate colonization with opportunistic pathogens generally harmless in normal individuals [53]. Despite activation of inflammatory responses mediated by the innate and cellular immune system, eradication of bacterial infection is impaired in CF lungs. Instead, bacterial infections induce a massive and chronic recruitment of neutrophils, which ultimately contributes to irreversible airway remodeling, observed as air trapping, bronchiolar obstruction, bronchiectasis, and loss of function [14].

### 2.4. Sterile Lung Inflammation in CF

While chronic infection is undoubtedly a major issue in CF pathology, it remains contested whether inflammation in CF only results from bacterial infection or is an intrinsic property of CFTR-deficient mucosa. Lung disease and inflammation is already observed in CF infants before bacterial colonization, suggesting that inflammation may precede infection in CF lung. Several reports argue that bacterial infections are indispensable to inflammatory responses, in CF humans [54] or CF pig [55]. However, unchallenged CF mutant mice show inflammation [56]. Also, in the CF ferret model, inflammation and tissue remodeling do not require previous bacterial infections [57]. Such sterile inflammation may be caused by functional abnormalities in CF myeloid cells, in particular macrophages [58, 59], dendritic cells [56], and neutrophils [60] or may be primarily related to abnormal cytokine signaling by CFTR-deficient airway epithelial cells. Several studies suggest that CFTR malfunction leads to overexpression of growth factors and proinflammatory cytokines as a cell-autonomous defect [61–63]

Due to the early onset of CF lung disease and its irreversible nature, it is clear that CF patients require early intervention therapy [32]. Excessive lung inflammation and tissue remodeling observed in CF may be an inherent property of CFTR-deficient lung mucosa. Therefore, it is important to establish the mechanisms involved in CFTR-related inflammatory responses and whether alleviation of inflammatory responses is beneficial in the management of CF lung disease [64].

## 3. Strategies of CF Therapy

CF lung disease is still the main cause of morbidity and mortality in CF despite intensive treatment [14]. Indirect pharmacological management focuses on anti-inflammatory agents [65, 66], antibiotics [67], and mucolytic agents [68]. Direct pharmacological management of CF disease intends to restore the functional expression of mutated CFTR at the plasma membrane by correcting its folding and gating defect [69]. In 2015, the FDA approved ORKAMBI® for homozygous F508del CFTR patients, comprising fifty percent of the CF population, the combination of CFTR corrector lumacaftor (VX-809) and potentiator ivacaftor (VX-770) in one pill. This therapy improves lung function in patients homozygous for the F508del mutation, although modestly and not in all patients [25, 70]. Therefore, further investigations are in progress to find more effective compounds [71, 72]. So far, the effects of correctors and potentiators on infections and the release of inflammatory mediators have not been broadly investigated in clinical studies. Rowe et al. showed that ivacaftor reduces *P. aeruginosa* isolated from CF patients carrying a CFTR gating mutation after 6 months treatment. However, the free neutrophil elastase and other inflammation markers like IL-1*β*, IL-6, and CXCL8 (IL-8) in sputum samples remained unchanged [73]. Therefore, anti-inflammatory, antibacterial, and other additional therapies are still important targets of investigation [74].

In summary, recent breakthroughs in the development of small-molecule compounds targeting the mutant CFTR protein have raised hope to find a cure for CF. However, the presently available CFTR correctors and potentiators are not sufficiently effective in a majority of CF patients, and the search for new compounds and additional therapies is still highly relevant. Though CF is considered monogenetic, the downstream responses to CFTR deficiency and responses to therapeutic intervention are highly variable in the population. The perfect corrector and potentiator combination tailored to the individual patient, supported by additional anti-inflammatory medication (personalized medicine), would likely be the best solution.

## 4. COPD Is Acquired CF?

Chronic obstructive pulmonary disease (COPD) is the 5th ranking cause of death worldwide. Usually, COPD is characterized by chronic bronchitis and emphysema [75]. Similar to CF, bronchiectasis [76] and peripheral airway thickening are also observed, similar to early and advanced CF lung disease [77]. Some individuals develop lung disease dominated by emphysema, while others exhibit chronic bronchitis. This heterogeneous phenotype likely reflects the contribution of multiple pathogenic mechanisms and the genetic heterogeneity of the population. Once COPD starts to develop, it tends to worsen over time, and so far its progress cannot be controlled effectively in most patients. The most prominent etiological factor leading to COPD is cigarette smoke, and also exposure to fumes, chemicals, and dust [78]. Although COPD and CF differ in primary cause, the spectra of the pathological events overlap considerably (Figure 1). Chronic bacterial infections with frequent exacerbations are observed, including colonization with the opportunistic pathogen *Pseudomonas aeruginosa*, which are also a hallmark of CF lung disease. However, the strains adapted to COPD lungs appear to differ from CF, suggesting that although similar mechanisms are involved, the luminal milieu in COPD differs from that in CF [79]. Both diseases are characterized by excessive mucus production and insufficient clearance, leading to lower airway obstruction and chronic neutrophilic infiltration. In CF and COPD, airway surface liquid (ASL) dehydration and viscous mucus secretion impair mucociliary clearance, causing chronic inflammation and facilitating recurrent infections [80]. A wide variety of proinflammatory mediators in COPD (like CXCL8, IL-6, and CCL18) overlap with CF-related mediators [74, 81]. There are also parallels on the cellular level which include goblet cell metaplasia, hyperplasia of myoblasts, and extensive extracellular matrix production [17].

The broad spectrum of common features and events observed in CF and COPD encouraged researchers to seek common factors for these diseases. Cigarette smoke decreases CFTR mRNA expression and reduces CFTR protein level through accelerated degradation, leading to impaired mucociliary clearance and depleted ASL in vitro and in vivo [82–84]. Mice treated with cigarette smoke show reduced CFTR activity that can be corrected by the cAMP agonist and phosphodiesterase inhibitor roflumilast, restoring CFTR activity [85]. Furthermore, macrophage phagocytosis and CFTR activity are impaired by cigarette smoke [86]. Similarly, CFTR-deficient macrophages are abnormal [59]. These are short-term and transient effects in normal cells, which do not explain the progressive inflammatory lung disease of COPD patients that stopped smoking. However, several reports show that the abnormal characteristics of COPD epithelial cells, including reduced CFTR expression in situ, persist in culture [87], probably due to a combination of genetic factors and epigenetic imprinting. Furthermore, COPD patients, smokers, and former smokers show signs of persistent reduced CFTR function in upper and lower airways, which may contribute to chronic lung disease [80]. Consequently, several authors described COPD as an “acquired CF” through e reduction of CFTR activity, suggesting that these diseases with different etiology have common therapeutics options [16, 17, 80, 85].

## 5. Strategies of Therapy in COPD

Based on the previous arguments, CFTR potentiators and correctors, such as those developed to enhance the activity of mutant CFTR, could be useful to treat COPD. However, small molecules designed to target mutant CFTR trafficking and gating may actually reduce activity of normal CFTR upon chronic treatment [88]. Nevertheless, further screening and testing of novel compounds in a personalized setting may lead to improvement of CFTR function and reduction of lung disease in COPD patients.

Chronic inflammation is recognized as the major pathophysiological mechanism of COPD progression, with molecular targets overlapping those of CF (e.g., IL-6, CXCL8, and CCL18) [74]. Thus, chronic airway inflammation remains an important therapeutic target in COPD management. It often persists after cessation of smoking, suggesting that apart from the role of CFTR also epigenetic changes in the resolution of inflammation likely play a role [89, 90]. Presently, several compounds targeting inflammatory responses in COPD are under investigation [91, 92], though none of these have been shown to be beneficial in COPD patients as yet.

Therefore, as in CF, COPD is a complex multifactorial disease, with large variation in the patient population due to largely undefined genetic and environmental factors. A personalized approach using multiple treatments is likely required, but a robust trial strategy is elusive. The advance of personalized 3D culture models, combining induced stem cell (iPSC) and gene editing [93] with microfluidics (“lung-on-a-chip”) technology [94] may allow us to proceed towards better treatment.

## 6. Epithelial EGFR/ADAM17 Axis: A Potential Therapeutic Target in CF and COPD Lung Disease

The design of anti-inflammatory agents aims mainly to decrease the neutrophil influx into the lung and concomitant inflammatory responses [8]. The importance of airway epithelial cells in inflammatory responses has been recognized for decades, but the signaling network is still under intense investigation [1, 95]. Airway epithelium serves as the first barrier and acts as defense against daily inhaled air pollutants and microbes by mucociliary clearance and secretion of a range of cytokines, cytokine receptors, growth factors, growth factor receptors, and antimicrobial peptides [1, 96]. Airway epithelial cells not only release inflammatory mediators to ASL but also signal to the underlying tissues (myocytes or fibroblasts). One of the mechanisms controlling paracrine and autocrine proinflammatory and profibrotic signaling in airway epithelium is the EGFR/ADAM17 axis.

A disintegrin and metalloproteinase 17 (ADAM17), also known as a tumor necrosis factor-*α* converting enzyme (TACE), is a transmembrane protein with proteolytic activity. It releases extracellular domains of its substrates, generally transmembrane proteins, to produce soluble bioactive signaling proteins, in a process called shedding. Mature ADAM17 consists of several functional domains (Figure 2), an extracellular metalloprotease domain (catalytic), a disintegrin domain, a membrane proximal domain (MPD) rich in cysteine residues, and a “conserved ADAM-seventeen dynamic interaction sequence” (CANDIS). Unlike other ADAM family members, it lacks an EGF-like domain [97–101]. These extracellular domains are connected to a transmembrane region and cytoplasmic tail with phosphorylation sites that likely are involved in ADAM17 activation or trafficking (Figure 2).

Ectodomain shedding mediated by the metalloprotease domain of ADAM17 provides a mechanism for initiation or inhibition of autocrine/paracrine signaling. So far, 76 proteins have been identified as substrates of ADAM17 [18]. They encompass membrane-bound cytokines (TNF-*α*), cytokine receptors (IL-6R, TNF-R), growth factors, in particular ligands of EGFR (TGF-*α*, AREG, EREG, HB-EGF, and epigen), adhesion proteins (L-selectin, ICAM-1), and transmembrane mucins (MUC-1). The shed soluble forms of these proteins are bioactive transducers of cell signaling via activation of cellular receptors on underlying neighboring cells (transactivation/paracrine activation), but they also are involved in activation of the shedding cells and neighboring cells (autocrine activation) (Figure 3).

Because most of the epidermal growth factor receptor (EGFR) ligands are cleaved by ADAM17, this sheddase has emerged as an important transducer of the airway epithelial autocrine and paracrine EGFR signaling (Figure 3). EGFR and ADAM17 are both involved in the broad spectrum of events that is characteristic of both CF and COPD lung disease, like excessive mucus expression [102–104], cytokine secretion [105], airway epithelial cell wound healing [106, 107], abnormal airway proliferation [108], maintenance of barrier integrity, and progressive lung tissue scarring [109]. They both are activated upon bacterial or viral infection and during inflammation [3, 5, 110]. While this is an effective and necessary response, it is suggested that exaggerated airway epithelial signaling in the chronic state may enhance inflammation and may lead to permanent damage to the lung structure.

EGFR functions as a sensor of airway epithelial integrity [111]. When cells have intact tight junctions, EGFR is not activated. But disruption of the epithelial cell integrity, either by mechanical injury or cytokine treatment (TNF-*α*/IFN-*γ*), leads to EGFR phosphorylation and concomitant inhibition of protein phosphatase 2A activity [112]. Cigarette smoke exposure of differentiated HBEC also leads to damage of the lung tissue observed as destruction of epithelial cell integrity, loss of E-cadherin/*β*-catenin complex, and disappearance of cilia [113]. This coincides with phosphorylation and perinuclear trafficking of EGFR [113] suggesting the importance of EGFR in maintenance of epithelial cell barrier integrity. The response of ADAM17 to loss of pulmonary epithelial cell integrity has been also shown by neuregulin-1 (NRG-1) shedding and concomitant activation of human epidermal growth factor receptor-2 (HER2) [114]. This raises the question whether and how EGFR and ADAM17 cooperate in sensing responses to airway injury.

### 6.1. EGFR and ADAM17 Are Important in Lung Development

Inactivation of ADAM17 by deletion of the zinc binding domain through homologous recombination in mice led to severely hypoplastic lungs at birth, reduced branching morphogenesis and alveolar development, impaired epithelial cell proliferation, and differentiation and delay in vasculogenesis [115]. Due to the early mortality of ADAM-KO mice, several alternative ADAM17 mouse models have been developed, with reduced activity (hypomorphic) or conditional mutants [116]. Conditional knockout ADAM17 mice can serve as a model to investigate the role of ADAM17 in organogenesis, in specific cell types, and in inflammatory responses and tissue remodeling [20, 117]. Induced dysfunction of ADAM17 (ADAM17flox/flox SPC-rtTA TetO-Cre) in developing lung epithelial cells reduced saccular formation, cell proliferation, and lung epithelial cell differentiation, but the mice were born without severe respiratory distress [19]. Knocking out the gene in mesenchymal cells using a Dermo1-Cre transgene did not produce a detectable phenotype [19]. This suggests that epithelial, but not mesenchymal, ADAM17 plays a prominent role in lung development.

Other studies show that conditional knockout mice (ADAM17flox/flox with R26Cre-ER) treated with human neutrophil elastase have attenuated goblet cell metaplasia in comparison to the wild-type mice [118], suggesting that ADAM17 is involved in injury-induced airway metaplasia. Similar to ADAM17-KO mice, EGFR-KO mice survive for up to 8 days after birth and suffer from impaired epithelial development in several organs, including the lungs, underlining the need of a functional EGFR/ADAM17 axis for proper functioning and development of lung epithelium [119].

### 6.2. ADAM17 and EGFR Crosstalk

**Figure 4:**
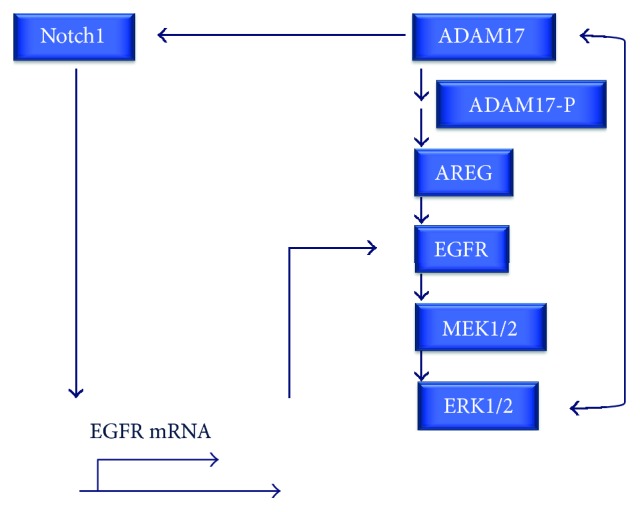
The ADAM17/EGFR axis. The EGFR/ADAM17 positive feedback loop likely involves activation of ADAM17 via the EGFR/MAPK pathway and direct interaction of ERK1/2 with ADAM17. Additionally, by cleavage of Notch1 from a non-small lung carcinoma cell line ADAM17 regulates transcription of EGFR mRNA and increases EGFR expression on the cell surface, providing another mechanism contributing to positive feedback regulation.

ADAM17 works in association with the tyrosine kinase receptor EGFR firstly by shedding most of its ligands, including AREG, HB-EGF, TNF-*α*, EPGN (epigen), and EREG (epiregulin) [18], resulting in activation of EGFR. In humans, only two EGFR ligands, EGF and betacellulin, are shed by ADAM10 [120, 121], a close relative of ADAM17 [18]. Crosstalk of ADAM17 and EGFR in inflammatory signaling transduction is further defined by the establishment of a positive ADAM17/EGFR feedback loop likely involving activation of ADAM17 via the EGFR/MAPK pathway [110, 122] (Figure 4). The exact molecular mechanism of this feedback signaling has not been firmly established. Moreover, ADAM17 regulates transcription of EGFR mRNA by cleavage of Notch1 and thus increases EGFR expression in a non-small lung carcinoma cell line [123], providing another positive feedback mechanism of the EGFR/ADAM17 axis (Figure 4).

In differentiated primary bronchial epithelial cells (HBEC-ALI), inhibitors of EGFR and of ADAM17 completely abolished ADAM17 substrate shedding as well as EGFR-dependent CXCL8 mRNA induction [87], illustrating the strong interrelationship between the key elements of the EGFR/ADAM17 axis (Figure 4).

### 6.3. The Role of ADAM17 and EGFR in Balanced Regulation of Lung Inflammation and Regeneration

ADAM17 is expressed in human bronchial epithelial cells in the large and small bronchi, in lung smooth muscle cells, lung muscular vessels, alveolar macrophages, perivascular leukocytes, and lung endothelial cells. It has been long recognized as an important regulator of lung tissue homeostasis [116, 124, 125], pro- and anti-inflammatory responses [18], and tissue regeneration [122, 126, 127].

The anti- and proinflammatory properties of the EGFR/ADAM17 signaling pathway are context and cell type dependent [18]. For instance, in airway epithelial cells, ADAM17 together with EGFR induces mRNA expression and protein release of CXCL8, a neutrophil chemotactic factor that promotes inflammation [7, 87, 105]. However, by shedding TNF receptor type 2 (TNFR2) [128], which antagonizes TNF-*α*, ADAM17 exhibits also anti-inflammatory properties [129]. Thus, it is likely that the type of stimulus and substrate selection determine the effect of the EGFR and ADAM17 activity [130–132].

EGFR/ADAM17 signaling in tissue regeneration encompasses wound healing [122], proliferation [126, 127], differentiation [19, 133], and cell migration [134]. Due to the involvement of EGFR/ADAM17 paracrine and autocrine signaling in several lung disorders [109, 116], modulation of ADAM17 and EGFR activation in both COPD and CF is important, to keep the balance between anti-inflammatory processes and promotion of inflammation, and also between regeneration and excessive tissue remodeling. Indeed, we recently found that cigarette smoke induced shedding of the ADAM17 substrate amphiregulin (AREG), and IL-6R was enhanced in differentiated bronchial cells in culture obtained from COPD patients compared to non-COPD, suggesting that epigenetic factors controlling the activity of the ADAM17/EGFR axis are affected in COPD [87].

### 6.4. Release of Cytokines, Growth Factors, and Mucins Depends on ADAM17/EGFR Signaling

External stress factors like oxidative stress, viral and bacterial toxins, and CS exposure activate the ADAM17/EGFR signaling pathway. Pathogens inhaled into the airways, or exposure to other stimuli, activate Toll-like receptors (TLR) [135–137] and G-coupled receptors (GPCR) [138] that crosstalk with downstream ADAM17/EGFR signaling. As a result of cigarette smoke extract exposure, secretion of downstream proinflammatory cytokines including CXCL8 [7, 139], growth factors (TGF*α*, AREG, and HB-EGF) [87, 105], cytokine receptor IL-6R [87], mucins (MUC5AC), and phosphorylated MUC1 is induced in an ADAM17/EGFR-dependent manner [103, 113, 140, 141]. This cascade of events involves mitogen-activated protein kinases (MAPK), MAPK1 (ERK1), MAPK2 (ERK2), and p38-MAPK [3, 142]. Upon activation of the ERK or p38 MAPK pathway, ADAM17 dissociates from an endogenous extracellular tissue inhibitor of metalloproteinase-3 (TIMP-3) [143-145], accumulates on the cell surface [146, 147], and induces release of TGF-*α* [146]. Acrolein, an active component of cigarette smoke, which induces MUC5AC mRNA in an ADAM17 and EGFR dependent manner, decreases TIMP-3 transcript levels, also suggesting a role of TIMP-3 in ADAM17 activation.

### 6.5. CFTR Deficiency Affects the EGFR/ADAM17 Axis

Several lines of evidence suggest that CFTR deficiency affects the activity of the EGFR/ADAM17 axis. F508del CFTR mutant mice respond differently from normal to airway injury by naphthalene [148]. A week after injury, we observed a significantly enhanced mRNA expression of amphiregulin (AREG) compared to normal [149]. AREG mRNA expression is dependent both on ADAM17 and EGFR activity in human bronchial epithelial cells [87], suggesting a link between CFTR deficiency and EGFR/ADAM17 activity during resolution of airway injury. Furthermore, inhibition of CFTR with a small molecule (inh-172) in NCI-H292 cells reportedly activates CXCL8 production in an EGFR/ADAM17-dependent way [61]. However, the relationship between ADAM17/EGFR signaling and CFTR deficiency is still poorly understood and may involve a variety of mechanisms [150].

Exaggerated responses mediated by the EGFR/ADAM17/MAPK pathway have also been reported in CFTR-deficient cells compared to CFTR expressing counterparts. The CFTR-deficient cell line IB3 produces more CXCL8 than an isogenic CFTR-expressing cell line. The CFTR-deficient cell line CuFi-1 produces more CXCL8 in response to heat-inactivated *P. aeruginosa* than a non-CFTR-deficient cell line (NuLi-1), and this involves EGFR phosphorylation and ERK1/2 activation [151]. Most of these studies use undifferentiated, submerged immortalized cell lines [152], compare genetically diverse cell lines (like CuFi and NuLi or IB3 and C38 cells) [61, 151, 153, 154], or use a CFTR inhibitor [61] with reported off-target effects [155]. Further studies in primary human bronchial epithelial cells in air-liquid interface culture, and models that allow comparison of genetically identical populations in parallel, including CFTR-deficient and corrected induced pluripotent stem cells (iPSC) and inducible CFTR-expressing CFBE cells, are currently performed to further support the notion that CFTR is involved in the activity of the EGFR/ADAM17 axis and may contribute to abnormal resolution of injury and inflammation in CF lung disease.

### 6.6. Redox Potential: A Possible Link between CFTR Deficiency and ADAM17/EGFR Signaling?

The intra- and extracellular redox potential changes in response to physiological processes and in pathophysiological conditions [156]. Reactive oxygen species (ROS), produced during cellular stress [74], are an important regulator of redox state and they are also involved in activation of the EGFR/ADAM17 signaling pathway, affecting TGF-*α* and AREG release and mucin expression [103, 104, 135]. Some studies point towards the role of NADPH oxidases (NOX), in particular dual oxidase 1 (DUOX1) [104, 135, 157] or dual oxidase 2 (DUOX2) [136], which produce ROS at an extracellular or possibly intravesicular domain. ATP-mediated DUOX1 activation involves a TGF-*α*/ADAM17/EGFR/ERK signaling pathway [157]. Recent studies indicate a role of DUOX1 in allergen-dependent SRC/EGFR activation in airway cells [158]. However, the mechanism by which ROS affect EGFR/ADAM17 signaling in intact airway cells is likely highly complex and still remains not well understood.

**Figure 5:**
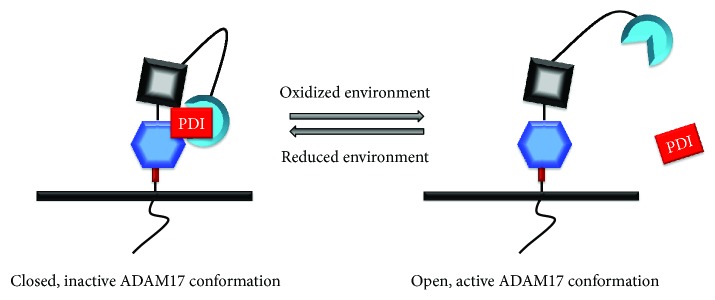
ADAM17 is a redox-sensitive protein. The ADAM17 membrane proximal domain (MPD), which is in close proximity to the active site, is sensitive to extracellular (or intravesicular) redox changes. This redox-sensitive ADAM17 activity is regulated by thiol-disulfide isomerization mediated by protein disulphide isomerase (PDI), an oxidoreductase sensitive to redox changes. PDI changes the disulfide bridge pattern and thus the conformation of the extracellular protease domain from an open active to a closed inactive state, by direct interaction with the membrane proximal domain (MPD), leading to the inhibition of ADAM17 proteolytic activity. Redox sensitive conformational changes likely make ADAM17 sensitive to the extracellular redox potential, which is dependent on glutathione (GSH/GSSH) and ROS.

ROS do not only affect receptors, phosphatases, and kinases in the ADAM17/EGFR pathway, but ADAM17 [159] and EGFR [160] themselves are redox-sensitive proteins. EGFR has intracellular cysteine residues in the active site that are targets of ROS and determine EGFR kinase activity, likely through association of EGFR with NADPH oxidase, NOX2 [160]. ADAM17 activity is regulated by thiol-disulfide isomerization in the extracellular MPD domain mediated by protein disulfide isomerase (PDI), an oxidoreductase sensitive to redox changes [161]. PDI, by direct interaction with the membrane proximal domain (MPD) [159], changes the disulfide bridge pattern and thus the conformation of the extracellular protease domain from open active to closed inactive state leading to the inhibition of ADAM17 activity (Figure 5) [101, 162]. Redox-dependent conformational changes likely make ADAM17 sensitive to the extracellular redox potential [159].

Since CFTR deficiency is thought to increase ROS levels [163], which would activate ADAM17 and EGFR [122] and inactivate protein phosphatases [164], we propose that redox signaling is a plausible link between EGFR/ADAM17 activity and CFTR deficiency. CFTR deficiency may change the extracellular redox potential to more oxidized in airway, because CFTR is involved in epithelial glutathione transport at the apical membrane [27, 165], which serves as a natural antioxidant [30]. This would in turn enhance the activity of ADAM17 through the mechanism illustrated in Figure 5. Polymorphisms in the glutathione pathway are modifiers of CF lung pathology, emphasizing the importance of this pathway [166]. Further studies are required to establish the relationship between glutathione transport, extracellular redox potential, and the activity of the EGFR/ADAM17 axis in CF airways. This can be achieved by application of fluorescent protein redox probes that can be expressed in different cellular compartments [156] in advanced airway culture models.

## 7. Role of Amphiregulin in EGFR/ADAM17-Dependent Lung Disease

One of the prominent ADAM17-dependent EGFR agonists produced ubiquitously by the human lung is amphiregulin (AREG) [167] by human airway epithelial cells [87, 105], smooth muscle cells [168], and fibroblasts [111]. AREG is also expressed by infiltrating and resident lung myeloid cells, including activated macrophages [169], eosinophils [170], dendritic cells [171], neutrophils [172], and mast cells [173]. Human AREG is synthetized as an N-glycosylated transmembrane precursor (50 kDa)[174] with a basolateral sorting motif [175] and shed mainly by ADAM17 [110]. Proteolytic cleavage of the AREG extracellular domain releases several AREG-soluble forms, predominantly an *α*-N-glycosylated 43 kDa form [174], which is one of the EGFR ligands, with lower affinity to EGFR than EGF and TGF-*α*.

### 7.1. AREG Is Induced in Lung Disease

AREG is involved in inflammation and repair responses through autocrine and paracrine activation of EGFR, and generally induced in lung disease [176]. In CF sputum samples, elevated levels of AREG have been shown in airway blood neutrophils [172]. In lung biopsies from asthma patients, more AREG is expressed than in healthy controls [167]. Other studies showed that in sputum of asthma patients AREG is upregulated only during an acute attack [177], suggesting its role in quick cellular responses to the triggers. Increase of AREG in sputum samples from children with asthma negatively correlates with lung function [178] and positively correlates with the number of eosinophils [179, 180].

Epithelial secretion of AREG in situ has not been investigated in CF and COPD in comparison to controls. However, Zuo et al. reported enhanced AREG expression in smoking-induced airway lesions [181], and we observed a stronger AREG shedding response to cigarette smoke in cultured airway cells from COPD patients compared to normal [87] suggesting a possible involvement in the progression of lung disease. Current studies are aimed at elucidating the role of CFTR deficiency in AREG shedding.

### 7.2. Regulation of AREG Transcription and Shedding

In vitro, AREG mRNA expression and protein release are induced upon exposure to different stress factors like histamine [167], diesel exhaust particles [6], cigarette smoke extract exposure [182], cigarette smoke [87], and rhinoviruses [3]. Also, AREG protein secretion is dependent on the EGFR/MAPK pathway in an airway epithelial cell line treated with particulate matter [183]. In differentiated primary airway cells, CS induction of AREG mRNA levels is abolished by ADAM17 and EGFR inhibitors, consistent with a prominent role of the EGFR/ADAM17 axis in AREG signaling and mRNA synthesis [87].

### 7.3. AREG Affects Mucus and Cytokine Secretion in Asthmatic Patients

In asthmatic patients, AREG produced by mast cells enhances mucus production [184]. AREG-dependent MUC5AC mRNA level induction has been shown upon exposure to particulate matter in NCI-H292 cells [185] and differentiated primary airway cells NHBE-ALI [177]. AREG-dependent secretion of MUC5AC, TGF-*β*1, and CXCL8 was also observed in epithelial cell line culture supernatants [179]. All these findings suggest an important role of AREG in mucus secretion and cytokine release in airway epithelial cells.

### 7.4. AREG in Paracrine Signaling and Tissue Remodeling

**Figure 6:**
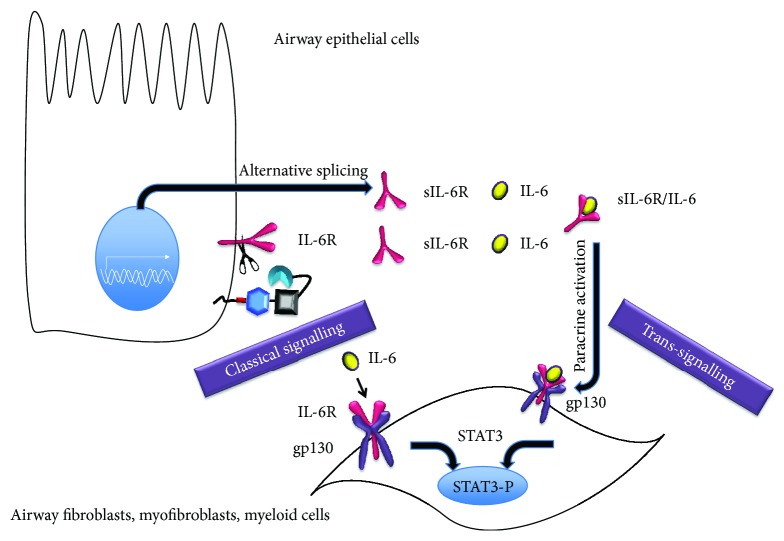
Classic and trans IL-6R signaling. In “classic” signaling membrane-bound IL-6R stimulated by secreted IL-6 associates with a homodimer of signal transducing glycoprotein (gp130) to activate downstream signaling molecules, including the ubiquitous transcription factor STAT-3. In trans-signaling, the soluble form of IL-6R (sIL-6R,) generated either by ADAM17 or by alternative mRNA splicing, binds to IL-6 and this IL-6/IL-6R complex activates gp130 on the airway epithelial cells (autocrine IL-6R trans-signaling) or underlying fibroblasts, myoblasts, or smooth muscle cells that do not express IL-6R (paracrine IL-6R trans-signaling), but do express gp130 to evoke activation of STAT-3 signaling.

In addition to its role in epithelial proliferation and differentiation (autocrine), AREG is also involved in fibrotic and inflammatory responses (Figure 3). Conditioned media from AREG-stimulated airway epithelial cells induced expression of CXCL8, VEGF, COX-2, and AREG in human airway smooth muscle cells, providing proof for paracrine crosstalk between epithelial cells and connective tissue in an AREG-dependent manner [168](Figure 6). Inhibition of AREG or EGFR in TGF-*β*1-stimulated lung fibroblasts diminished AREG-dependent fibroblast proliferation, expression of *α*-smooth muscle actin and collagen [186], strongly suggesting a role of EGFR/ADAM17/AREG signaling in pulmonary fibrosis in vivo. AREG stimulation also induces airway smooth muscle cell proliferation, which leads to airway remodeling in vivo [180].

These data together suggest that exaggerated and chronic AREG release may contribute to mucus plugging, excessive inflammation, and tissue remodeling in CF and COPD. Therefore, the EGFR/ADAM17/AREG signaling pathway is a potential therapeutic target to regulate both inflammation and lung cell proliferation in COPD and CF. However, AREG is only one of the many players involved in the autocrine and paracrine signaling leading to lung pathology. There is no available literature presenting intervention in AREG function in clinical trials.

## 8. Role of IL-6R in Human Lung Inflammation and Tissue Regeneration

Another prominent ADAM17 substrate involved in lung disease is the IL-6 coreceptor IL-6R. In “classic” signaling, this transmembrane receptor type I binds to IL-6, which evokes an association with a homodimer of signal transducing glycoprotein, gp130. This trimer dimerizes to form a hexameric complex composed of IL-6, IL-6R, and gp130 [187] (Figure 6). Alternatively, the extracellular domain of IL-6R can also evoke a trans-signaling cascade, when it is shed by ADAM17, producing a soluble form (sIL-6R) (Figure 6) [188] or ADAM10 [188–190]. sIL-6R can be also generated by alternative splicing of IL-6R mRNA [191–193]. However, data suggest that shedding rather than the alternative splicing takes part in trigger-induced generation of the sIL-6R [128]. Indeed, we observed that in primary bronchial epithelial cells in air liquid culture, cigarette smoke enhanced ADAM17-dependent sIL-6R shedding but not the production of the alternatively spliced mRNA [87]. Both the alternatively spliced form and the shed form of sIL-6R can create functional complexes with IL-6 (IL-6/sIL-6R) [194], mediating a transfer of stress responses from epithelial cells to underlying mesenchymal and myeloid cells, involved in tissue remodeling and inflammation (trans-signaling, Figure 6).

IL-6 activated membrane bound IL-6R has anti-inflammatory, antiapoptotic, and regenerative properties [128, 195–198]. In contrast, trans-signaling mediated by IL-6/sIL-6R complexes binding to gp130 is thought to maintain inflammation and promote inflammation-associated cancer [128, 195, 199, 200] (Figure 6).

Importantly, IL-6R expression is not ubiquitous, but restricted to specific cell types, such as lymphoid cells, hepatocytes, and airway epithelial cells [4, 128, 201, 202]. However, cells that do not express IL-6R but do express gp130 can still transduce IL-6 signals via binding of IL-6/sIL-6R complexes in trans. Thus, smooth muscle cells and endothelial cells are responsive to IL-6 through IL-6/sIL-6R trans-signaling provided by epithelial cells [203, 204] (Figure 6).

The role of IL-6R in CF and COPD has not been studied in detail. In COPD patients, elevated levels of IL-6R have been observed in peripheral blood leukocytes [205] and sputum samples [206]. Recently, genetic variants of IL-6R have been linked with lung function [207] and COPD severity [208]. In contrast to elevated IL-6R levels in sputum of COPD patients [207], the levels of sIL-6R in BALF from CF patients were not different in comparison to control, possibly due to enhanced degradation of sIL-6R by serine proteases [209]. Furthermore, it is not evident what the impact of luminal IL-6R on the activity of the subepithelial tissue is, which is separated from the lumen by tight junctions. Shedding of IL-6R from epithelial cells occurs mainly towards the basal side where it can engage in paracrine and autocrine signaling (Figure 6) [4, 87]. Inhibition of sIL-6R by intraperitoneal injection of a recombinant decoy receptor (gp130Fc) attenuates pulmonary fibrosis, whereas activation of IL-6 trans-signaling in cell lines enhances fibroblast proliferation and extracellular matrix protein production [210], which are the hallmarks of the pulmonary fibrosis progression.

Together, these data show the importance of IL-6R trans-signaling in inflammation and lung remodeling and offer possibilities for therapeutic interventions [14].

### 8.1. Species Specificity of IL-6R Signaling

Murine models have been invaluable in the study of inflammatory disease, but should be analyzed with caution. Species differences in IL-6R mediated signaling have been observed. Human IL-6 stimulates human and murine cells, whereas murine IL-6 only stimulates murine IL-6R signaling [211]. Because of this species specificity, transgenic mice expressing human sIL-6R from a liver promoter did not bind the endogenous murine IL-6, and as a consequence the transgenic animals do not have a transgene specific phenotype [211, 212]. Garbers et al. reported that human IL-6R is a substrate of human ADAM17, but murine IL-6R is a substrate of murine ADAM10 [213]. However, Schumacher et al. subsequently revealed that trigger-induced shedding of both human and mouse IL-6R is mediated by murine ADAM17, but constitutive release of IL-6R is largely mediated by mADAM10 in mice [190]. Additionally, in humans, but not in mice, the sIL-6R can be generated by translation from an alternatively spliced mRNA [190, 214]. Of note, the soluble gp130 form, which circulates in human plasma, blocks IL-6R trans-signaling responses and does not show species specificity, meaning that it interacts with human and mouse sIL-6R complexes [215]. All of this has implications in the extrapolation of IL-6R data obtained in mouse models to human, but is consistent with a prominent role of EGFR/ADAM17 sIL-6R trans-signaling in human lung pathology.

### 8.2. Dual Control of the STAT3 Transcription Pathway by the EGFR/ADAM17 Axis

**Figure 7:**
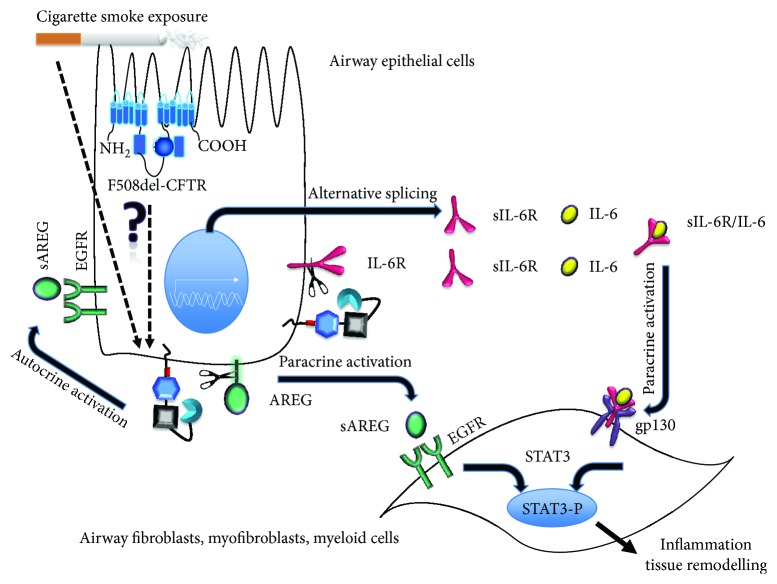
ADAM17/EGFR signaling an important player in CF and COPD lung disease. In airway epithelial cells in culture, both CFTR deficiency [61] and cigarette smoke exposure [87] cause enhanced activity of the EGFR/ADAM17 signaling cascade, and thus enhanced release of proinflammatory cytokines and growth factors that are ADAM17 substrates and signaling molecules that are downstream of the EGFR/MAPK pathway (Figure 1) including the proinflammatory CXCL8 (IL-8). In chronic lung disease, this could contribute to mucous metaplasia, inflammation, and tissue remodeling. A potential link between CFTR deficiency, COPD, and EGFR/ADAM17 activity (dashed arrows) is the extracellular redox potential, which is in part dependent on CFTR-related glutathione transport [30, 165, 166], and which in turn regulates ADAM17 activity (Figure 5). In COPD, CS induced long-term downregulation of CFTR expression [16, 47, 80] can have the same long-term effect, in addition to the acute oxidative stress caused by cigarette smoke. In this figure, we focus on two canonical ADAM17 substrates: proinflammatory IL-6 receptor (IL-6R) and growth factor amphiregulin (AREG). AREG is proteolytically cleaved by ADAM17 from airway epithelial cells and activates EGFR in the airway epithelial cells and on the underlying fibroblasts. IL-6R trans-signaling requires ADAM17-mediated shedding of the extracellular domain or alternative mRNA splicing. The soluble sIL-6R binds to IL-6 creating IL-6/sIL-6R complexes that trans-activate gp130 on the underlying myofibroblasts and myeloid cells. Signals transduced by AREG and IL-6R shed from airway epithelial cells converge in combined activation of the transcription factor STAT3 in myofibroblasts and smooth muscle cells. STAT3 is involved in fibrotic responses and inflammatory lung disease, and a modifier of CF lung disease [221]. Based on available literature cited in text, enhanced EGFR/ADAM17 signaling may contribute to hyperinflammation and epithelial metaplasia, fibroblast and smooth muscle cell activation, angiogenesis, and net deposition of extracellular matrix (tissue remodeling) observed in COPD and CF lung disease.

IL-6R-mediated signaling leads to activation of the transcription factor STAT3 [216], ERK [217], and PI3K [201], linking this signaling pathway with the EGFR/ADAM17 axis. STAT3 is also activated through a parallel pathway in trans-signaling, which involves shed EGFR ligands [218](Figure 7). The STAT3 pathway is involved in tissue repair [198], carcinogenesis [9, 219], and immune responses [220]. Notably, STAT3 was found to be a modifier gene of cystic fibrosis lung disease [221], and enhanced STAT3 phosphorylation was observed in lung tissue from smokers and COPD patients [222]. Consequently, a disturbance in the control of the complex EGFR/ADAM17/STAT3 pathway would likely play a role in Cystic Fibrosis and COPD chronic lung disease.

## 9. ADAM17 Phosphorylation and Trafficking

The serine- and threonine-rich ADAM17 cytoplasmic tail [112] has three phosphorylation sites that have been proposed to activate ADAM17: Thr735, Ser791, and Ser819 (Figure 2). Pro-ADAM17 and mature ADAM17 are phosphorylated at Thr735 under resting conditions, but phorbol ester (PMA) treatment further increases phosphorylation at this site [223]. ERK 1/2 and p38 MAP kinase phosphorylate ADAM17 at Thr735 [126: Diaz-Rodriguez, 2002 #237] [224], and activate ADAM17 proteolytic activity, leading to shedding of IL-6R and TGF-alpha [126]. Stimulation of ADAM17 increases its phosphorylation without changing the total protein level, suggesting that phosphorylation plays a role in the regulation of the proteolytic activity of ADAM17. However, mutation of the phosphorylation sites individually or in combination and even removal of the whole cytoplasmic tail has no significant effect on stimulated shedding in cell models, suggesting that phosphorylation may be a minor regulatory mechanism of ADAM17 proteolytic activity, or that it plays a context and cell type dependent role in intracellular transport, processing, and maturation [112, 121, 225–227].

### 9.1. The Role of ADAM17, AREG/IL-6R, and EGFR Trafficking in Cellular Signaling

The majority of EGFR signaling is believed to occur at the plasma membrane. However, many studies show that upon ligand-dependent activation, EGFR is rapidly internalized into endosomes, where hypothetically it may continue to signal [228, 229]. Up till now, the triggered endocytosis of EGFR has been interpreted as signal attenuation, due to lysosomal degradation [230]. However, recent reviews elaborate on the role of EGFR endocytosis and recycling in active signal transduction [10, 230–233]. How triggered intracellular trafficking of EGFR relates to the regulation of the EGFR/ADAM17 axis has not been explained in detail. So far, these studies largely focus on deregulated EGFR trafficking in the context of carcinogenesis. However, EGFR trafficking in CFTR deficiency and its effect on the signaling cascade has not been investigated.

Most reports assume that ADAM17 cleaves and sheds it substrates at the extracellular membrane. However, ADAMs and its substrates have been localized in various subcellular compartments like lysosomes [234], endosomes [175, 235], and exosomes [233]; thus, it is difficult to identify an exact site of ADAM17 mediated shedding and how it interacts with EGFR [236].

Doedens et al. proposed that endocytosis from the cell surface is a pre-requisite for ADAM17 catalytic activity [237]. Lorenzen et al. suggested that ADAM17 and its substrate meet in the ER/Golgi pathway and that the prodomain does not interfere with the enzyme-substrate interaction [100]. Xu et al. showed that ADAM17 is localized at the cell surface as a dimer and associates with tissue inhibitor of metalloproteinase-3 (TIMP-3) [126]. Upon activation with PMA or anisomycin, the amount of ADAM17 at the cell surface increases, followed by dissociation of ADAM17 from TIMP-3, and ADAM17 dimers convert to monomers. According to the authors, these monomers correspond to the active form of ADAM17 and are predominantly found in cytoplasm [147], suggesting that the monomers are internalized, and shedding may occur intracellularly. Also, recent reports suggest that ADAM-mediated cleavage occurs from an intracellular vesicular pool in several cell types. Triggered shedding of FasL by ADAM10 and ADAM17 by T-lymphocytes involves trafficking of proteases and substrates to an intracellular membrane raft compartment [234]. Moreover, two proteins that regulate ADAM17-dependent shedding of EGFR ligands, annexins, and a phosphofurin acidic cluster sorting protein 2 are close to ADAM17 in the intracellular vesicular compartment [235] as shown by proximity ligation assay (PLA). Consistent with this, we have shown by PLA that upon CS exposure ADAM17 and ADAM17-P appear in close proximity with its substrates AREG and IL-6R in the intracellular compartment in ALI-HBEC, whereas under basal condition ADAM17 or ADAM17-P substrate complexes were infrequent [87]. Together, reports from various cellular models suggest that the shedding process may occur in an intracellular compartment and that triggered activity involves active trafficking of ADAM17 and its substrates. It remains to be established whether ADAM17 sheds its substrate in intracellular vesicles followed by secretion or whether the ADAM17/substrate complexes in these vesicles need to be transported to the membrane first in order to deliver ADAM17 and its substrate for cleavage. In both cases, a vesicle trafficking and membrane fusion event is involved, which may be subject to regulation, adding another level of complexity.

A further aspect of the relationship between EGFR/ADAM17 signaling and membrane trafficking concerns the production and intercellular exchange of exosomes. Higginbotham et al. showed the importance of paracrine exosomal AREG-mediated signaling in breast cancer cells [233]. Recipient LM2-4175 cells rapidly take up AREG-containing exosomes in an EGFR-dependent manner and enhanced invasion of LM2-4175 cells through matrigel [233]. Also, EGFR- and ADAM17-containing exosomes have been described [238, 239]. Such exosomes are considered important in the resolution of tissue injury and inflammation, presumably because they allow delivery of functional signaling complexes from triggered cells to neighboring cells. While most available studies address the role of exosomes in the progression of cancer, their role in chronic lung disease and possible implications for future treatment is under study [240].

## 10. Summary and Conclusions

Several lines of evidence, discussed above, suggest that the ADAM17/EGFR axis and downstream regulatory pathways are hyperactive in CF and COPD chronic lung disease, promoting inflammation and tissue remodeling by shedding EGFR binding growth factors and proinflammatory agonists from airway epithelial cells. This may contribute to inflammation, epithelial metaplasia, fibroblast and smooth muscle cell activation, and net deposition of extracellular matrix. Taken together, we propose that pathology-driven trans-signaling at least in part depends on airway epithelial AREG and IL-6R shedding. Importantly, signals transduced by shed AREG and IL-6R from airway epithelial cells may converge in activation of the transcription factor STAT3 in lung fibroblasts, myofibroblasts, and smooth muscle cells (Figure 7). Moreover, unbalanced ligand shedding towards the submucosa in CF and COPD and likely other chronic lung disease will affect the activity of resident and infiltrating myeloid cells, including dendritic cells, macrophages, and neutrophils.

While evidence in cellular and animals models is compelling, direct evidence for EGFR/ADAM17 hyperactivity from patient lung tissue *in situ* is scarce. This is in part due to a lack of appropriate airway material for analysis, especially from early disease and healthy controls. In a recent study, Zuo et al. show enhanced AREG expression in biopsies from smokers lungs, associated with remodeling lesions [181], consistent with enhanced EGFR activity as shown in primary cells in culture [87, 181]. However, successful pharmacological interventions in the EGFR/ADAM17 pathway have not been reported to our knowledge. Activation of CFTR activity may help to reduce EGFR/ADAM17 activity and resolve COPD pathology [85], but no clinical evidence for this is yet available. Targeting EGFR/ADAM17 in CF patients has not been attempted. Conversely, it would be important to study patients treated with CFTR-targeted medication for evidence of reduced activity of the EFGR/ADAM17 axis in biomarker and biopsy studies.

Genetic analysis of the COPD and CF populations, aiming at identification of genes that determine the pathology (“modifier genes”) would also provide important evidence for the involvement of the EGFR/ADAM17 axis. So far, as already cited above, STAT3, controlled by the EGFR/ADAM17 axis (Figure 7), is a modifier of CF lung disease [221]. IL-6R, a prominent ADAM17 substrate linking the axis to STAT3 in trans-signaling, is a modifier of COPD as well as asthma [207, 208]. So far, polymorphisms associated with the ADAM17, AREG, or EGFR genes have not been directly linked to chronic lung disease, but that may be due to limitations of the studies.

A possible mechanistic link between EGFR/ADAM17 activity, CF, and COPD is suggested by the observation that CFTR activity is diminished in COPD airways. Reduced CFTR activity interferes with glutathione transport in CF airways, adding to oxidative stress, which would activate the EGFR/ADAM17 axis both on CF and COPD. Further studies in vivo and in vitro are required to establish this.

Interventions in the EGFR/ADAM17 pathway may reduce CF and COPD lung pathology. However, since long-term systemic delivery of available EGFR and ADAM17 inhibitors likely causes undesirable side effects, novel methods to control this pathways will likely be required. Studies of stress-induced dynamic trafficking of the membrane proteins involved in the EGFR/ADAM17 axis in advanced 3D airway culture models may allow the development of novel modulators that will allow a more targeted approach to suit the requirements for treatment of CF and COPD lung disease.
